# Production and impact of Italian researchers in physical—Sport education and sport pedagogy

**DOI:** 10.3389/frma.2025.1478317

**Published:** 2025-05-06

**Authors:** Francesca D'Elia, Tiziana D'Isanto, Sara Aliberti, Rosario Ceruso, Giovanni Esposito, Gaetano Raiola

**Affiliations:** ^1^Department of Human, Philosophical and Education Sciences, University of Salerno, Salerno, Italy; ^2^Research Center of Physical Education and Exercise, University of Pegaso, Naples, Italy; ^3^Department of Neuroscience, Biomedicine and Movement, University of Verona, Verona, Italy; ^4^Department of Political Science and Communication, University of Salerno, Salerno, Italy

**Keywords:** scientific production, scientific disciplines, Scopus, sports science, citations

## Abstract

**Introduction:**

In Italy, legislation in Exercise and sports sciences (ESS) had significant progress, particularly in physical and sport education, as well as sport pedagogy for compulsory teaching of Physical education by master's degree' specialist teachers, for the new profession of kinesiologist and, first the reform of Italian Constitution about social and educational value of movement and sport. The aim of this study was to measure the specific scientific output in ESS and its impact on the scientific community.

**Methods:**

Data on global scientific production related to the keywords “Physical education,” “Sport education,” and “Sport pedagogy” were extracted from the Scopus database, including total citations and h-index. Trends among Italian researchers were also examined. Data on the overall scientific output and specific trends of Italian researchers were extracted, along with total citations and h-index. The trends of total and relative metrics (citations and h-index) for the period 2020–2023 were evaluated using Spearman's correlation.

**Results:**

Analysis of global scientific production on Scopus revealed 31 ESS faculty members (7 full professors, 15 associate professors and 9 researchers) for “Sport Education” keyword, 11 ESS faculty members (2 full professors, 5 associate professors, and 4 researcher) for “Sport Pedagogy,” and 18 ESS faculty members (7 full professors, 7 associate professors and 4 researcher) for “Physical Education.” Less than half of these researchers are directly framed with ESS. However, despite this minority representation, significant positive correlations emerged between total citations and total H-index for the period 2020–2023 (*rs* = 0.83), relative citations and relative H-index (*rs* = 0.61), relative citation and total H-index (*rs* = 0.32).

**Conclusions:**

The positive correlation found between relative citations and the total h-index indicated these scholars had a greater impact with studies relevant to the identified keywords compared to other general topics. The analysis also highlighted the lack of international impact of Italian research in the educational and didactic aspect of ESS, particularly for “Physical Education.”

## 1 Introduction

In Italy, research on scientific production in Exercise and Sports Sciences (ESS) began to develop only recently. Previous studies (D'Isanto et al., 2023, 2024) examined the correspondence between the titles of the most cited scientific works on Google Scholar by Italian scholars in the academic disciplines (AD) “Methods and didactics of motor activities” (code M-EDF/01) and “Methods and didactics of sports activities” (code M-EDF/02) and their respective descriptors. The results showed that the scientific production of researchers in the M-EDF/02 sector was more aligned with the biomedical area, while that of re-searchers in M-EDF/01 was more aligned with the pedagogical area (D'Isanto et al., 2023). This trend was attributed to the significant role of the AD defined by the norm as “related,” i.e., the AD that determined the scientific identity of scholars in ESS (Gazzetta Ufficiale, 2000).

The academic recognition of specific movement-sports knowledge dates to 2000, with the establishment of the two AD, that, subsequently, were grouped into 9 competition sectors (ARF), later reduced to 2 (Gazzetta Ufficiale, 2011, 2015). Later, the Ministry of University allocated these ADs to two CUN areas (6—Medical Sciences; 11—Historical, Philosophical, Psychological, and Pedagogical Sciences), creating an epistemological duality based more on regulations than on scientific criteria. To address this issue, the CUN (2016) identified 2,600 keywords to align the Italian classification model, with international standards (Scopus, Web of Science).

Subsequently, he proposed replacing ADs with disciplinary groupings based on fixed and mobile keywords, following the ERC model (CUN, 2018).

This reorganization, integrated in 2023, led to the creation of 190 scientific disciplinary groups (SDG), aligning Italy with EU requirements under the National Recovery and Resilience Plan. With DM No. 639/2024 (Gazzetta Ufficiale, 2024), the two ADs of ESS were incorporated into the new SDG “Physical Exercise and Sport Sciences” (06/MEDF-01), ensuring an autonomous scientific identity.

Twenty-five years after the introduction of ESS into Italian universities, it is useful to measure the impact of the scientific production of Italian scholars within the inter-national scientific community. This can be done using the basic model of scientific knowledge classification adopted internationally, characterized by domains, fields, and subfields (Rivest et al., 2021). This model led to the creation of the global ranking of the Top 2% Scientists, developed by Stanford University (Ioannidis, 2022, 2023; Ioannidis et al., 2019, 2020, 2021) in collaboration with Elsevier and the Scopus database (2023). For each scientist, their field of activity, ranking, citations, and h-index are indicated. The resulting composite index allows for measuring the citation impact of each researcher and specific work, applying corrective factors related, for example, to self-citations, single author works, and bibliometric dynamics of each scientific area. However, the database does not provide disaggregated citation data by country for scholars active in ESS, making it difficult to evaluate the overall impact of specific research conducted by Italian researchers in ESS.

Specific studies (Raiola et al., 2018; Raiola, 2020) identified keywords for the educational-didactic component of ESS, namely Physical and Sport Education and Sport Pedagogy. These three keywords are included in the functionalities of the Scopus database and were used to aggregate keywords in individual subfields by Ioannidis et al. for the top 100,000 scientists and by Rivers et al. for identifying scientific knowledge. Specifically, using Scopus Elsevier's “Research Discovery” function, it is possible to extract data on scientific production and its impact on the international scientific community, applying the specific Italian classification of scientific knowledge through the scientific-disciplinary descriptors of the AD that contain the specific keywords (D'Elia et al., 2023).

A previous study (Raiola et al., 2024) analyzed the impact of the scientific production of Italian ESS scholars, focusing on the subfield “Sport Sciences” using some characteristic keywords of the subfield. From this study, data extracted from the Scopus database for the period 2017–2022 included information on articles, citations, and the h-index of the top 200 Italian scholars. It emerged that the overall production and impact of the scholars followed the trend of production and impact related to the subfield's keywords, indicating proportionality in overall research compared to that focused on specific keywords. Another study (under review) accurately evaluated the impact of the scientific production of ESS scholars weighted for authorship, with specific weight attributions for individual authors extrapolated from the authorship of the articles, revealing positive and significant relationships between citations and the total weighted h-index compared to the same indicators related to the subfield “Sport Sciences,” both among corresponding and cross-indicators.

### 1.1 Problem

In Italy, legislation on ESS has seen significant developments, particularly concerning physical and sports education, as well as sport pedagogy for the mandatory teaching of physical education by specialized teachers (Altavilla, 2023; D'Elia, 2019a,b, 2023) with a master's degree, the new profession of kinesiologist, and especially for the reform of the Italian Constitution on the social and educational value of movement and sport. The scientific community has contributed through scientific production and its related impact. Currently, in Italy, there are still no systematic studies that evaluate the citation impact of scholars in ESS within the field of sport pedagogy.

### 1.2 Objective

Therefore, the objective is to measure the consistency of the impact of the scientific research of Italian scholars in ESS for the reference period 2020–2023 for the specific subfield of sport pedagogy, which includes the three keywords: “Physical Education,” “Sport Pedagogy,” and “Sport Education” compared to the impact of their entire scientific production.

## 2 Methods

The analysis of the scientific production of Italian scholars concerning the three previously identified keywords within the subfield required several processing phases.

(1) All structured ESS authors from the two ADs in the CINECA database were considered. Next, those who had publications relevant to the identified keywords, verifiable through the Researcher Discovery function of Scopus, were selected. The search was specifically configured to request the software to return only Italian authors who had produced works associated with each keyword, to include them in the analysis. The software provided up to a maximum of 200 authors associated with the searched keyword for each analysis.
(2) After identifying Italian ESS scholars through the search for each keyword, the following bibliometric parameters for the period 2020–2023 were collected:
  - Total Citations: Includes all citations obtained by an author, regardless of the specific field, including studies not related to the motor-sports field.
  - Relative Citations: Refers only to publications related to one of the three keywords.
(3) Final Phase: The weighted total h-index and the weighted relative h-index related to each keyword for each ESS scholar were calculated. The relative h-index was calculated considering each of the identified keywords. For each keyword, relative citation indices were determined, resulting in a term-specific h-index. Then, for each scholar, the relative values for the number of articles and citations associated with the keywords were summed. In addition, for each author, the values of the relative h-indexes corresponding to the keywords were summed, and finally, the total obtained was divided by the total number of relative h-indexes to obtain the numerical quotient of the relative h-index. Finally, the total number of articles, citations and h-index were tabulated for each author, together with the corresponding relative keyword values extracted from the database. In this way, it was possible to obtain both an overall measure of scientific production and its impact and a specific analysis based on the keywords detected.

### 2.1 Statistical analysis

Descriptive statistics (mean ± SD; %) were used to summarize the data for the different variables. The Shapiro-Wilk test revealed non-normal distribution, providing an initial insight into the nature of the data. To assess the trend between the total and relative metrics for the period 2020–2023, Spearman's correlation was used, considering the following interpretation of correlation coefficients: weak (0.10–0.39), moderate (0.40–0.69), and strong (≥0.70). Statistical Package for Social Science software (Version 27.0, IBM SPSS Statistics, Chicago, IL, USA).

## 3 Results

From the analysis of national scientific production on Scopus from 2020 to 2023, categorized by author role, interesting results are showed in Table 1. Full professors exhibited the highest total citation count (1429.3 ± 1229.8) and H-index (10.7 ± 5.1), indicating a greater overall impact. Associate professors followed, with moderate citation and H-index values, while research scientists showed the lowest values across all metrics. Relative citation and relative H-index values were more balanced among the three groups, suggesting differences in research visibility and productivity.

**Table 1:** Analysis of national scientific production on Scopus divided by author role.

**Authors information**	**Total citation 2020–2023**	**Total H-index 2020–2023**	**Relative citation 2020–2023**	**Relative H-index 2020–2023**
Full professors (*n* = 10)	1429.3 ± 1229.8	10.7 ± 5.1	61.7 ± 41.8	2.3 ±0.9
Associate professors (*n* = 18)	871.4 ± 827.6	9.1 ± 3.4	56.7 ± 56.6	1.7 ± 0.8
Research scientists (*n* = 10)	427.6 ± 314.3	6.9 ± 2.5	31.4 ± 30.6	1.6 ± 0.8

For the keyword “Sport Education,” 31 M-EDF structured scholars were identified at the national level, with the majority being associate professors, followed by researchers and full professors. At the international level, only five M-EDF structured scholars were classified, primarily full and associate professors. For the keyword “Sport Pedagogy,” 11 structured M-EDF scholars were identified at the national level, with most being associate professors, followed by researchers and full professors. Internationally, only four M-EDF structured scholars were classified, mainly associate professors. For the keyword “Physical Education,” 18 structured scholars were identified at the national level, with the majority being either associate or full professors. However, at the international level, no scholars were classified under this keyword. A detailed description is provided in Table 2.

**Table 2:** Distribution of M-EDF structured scholars by keyword, academic level, and position (national and international).

**Keyword**	**Level**	**M-EDF structured scholars**		
		**Full professors**	**Associate professors**	**Researchers**
Sport education	National	7 (22.6%)	15 (48.4%)	9 (29%)
	International	2 (40%)	2 (40%)	1 (20%)
Sport pedagogy	National	2 (18.2%)	5 (45.5%)	4 (36.4%)
	International	0 (0.0%)	3 (75%)	1 (25%)
Physical education	National	7 (38.9%)	7 (38.9%)	4 (22.2%)
	International	0 (0.0%)	0 (0.0%)	0 (0.0%)

**Figure 1:**
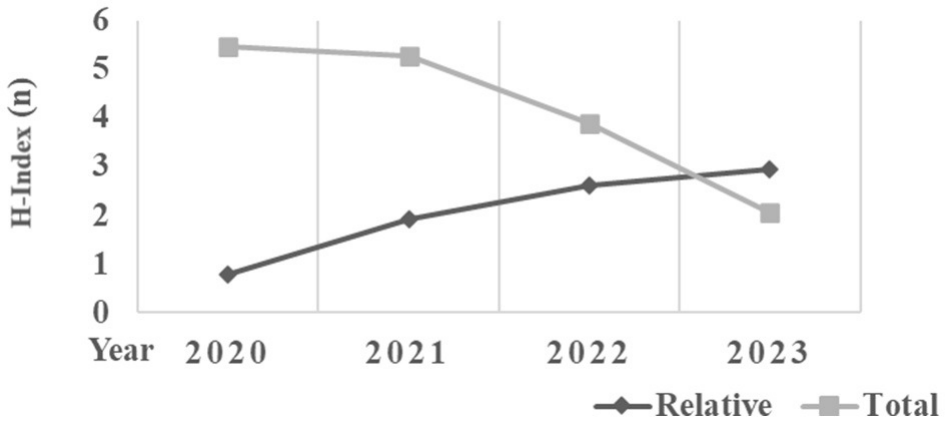
Trends of total and relative citations for the 3 keywords by year.

Figure 1 showed a graphical representation of both total and relative h-index trend for the 3 keywords (x-axis) by year (from 2020 to 2023; y-axis). While the relative h-index tends to increase gradually by year, the total h-index tends to decrease gradually.

**Figure 2:**
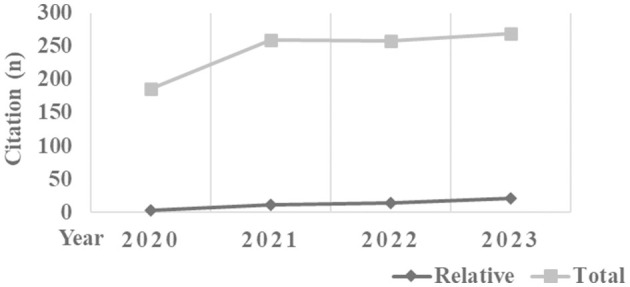
Trends of total and relative h-index for the 3 keywords by year.

Figure 2 showed a graphical representation of both total and relative citation trend for the 3 keywords (x-axis) by year (from 2020 to 2023; y-axis). While the total citations tend to increase gradually by year, the relative citations tend to increase.

To assess the existence of a trend over the 2020–2023 period between the metrics considered, a correlation analysis of the bibliometric parameters was conducted. Significant positive correlations emerge between total citations 2020–2023 and total H-index 2020–2023 (*rs* = 0.83), between relative citations and relative h-index (*rs* = 0.61) and finally between relative citations and total H-index (*rs* = 0.32). A detailed description is shown in Table 3.

**Table 3:** Correlation between bibliometrics values in 2020–2023 period.

**Correlations**		**Relative h-index 2020–2023**	**Total H-index 2020–2023**	**Total citation 2020–2023**	**Relative citation 2020–2023**
Spearman's rho	Relative h-index 2020–2023	1.000			
	Total H-index 2020–2023	0.184	1.000		
	Total citation 2020–2023	0.111	0.835^**^	1.000	
	Relative citation 2020–2023	0.616^**^	0.329^*^	0.305	1.000

* Correlation was significant at the 0.05 level (2 tails).

** Correlation was significant at 0.01 (2 tails).

## 4 Discussion

This study aimed to evaluate the impact of scientific research by Italian scholars within the ESS in the field of sports pedagogy, through three keywords: “Physical education,” “Sport Pedagogy,” and “Sport Education,” in relation to their entire scientific output for the period 2020–2023. The data analysis revealed that, within the group of 200 scholars returned by the Scopus database, only 39 scholars from the two ESS ADs addressed this topic. Nationally, the highest number of associated scholars was identified with the keyword “Sport Education,” with 31 scholars, including 15 associate professors, 7 full professors, and 9 researchers. This might indicate greater interest or a higher scientific production in this specific subfield. The following were “Physical Education” with 18 scholars and “Sport Pedagogy” with 11 scholars. Internationally, however, the number of identified scholars was significantly lower. For the keyword “Sport Education,” 5 scholars were identified, “Sport Pedagogy” had 4 scholars, while no scholars were identified for “Physical Education.” This might suggest lower visibility or competitiveness of Italian research in the international context for these specific areas of study. The larger number of scholars using “Sports Education” may indicate the growing recognition of its importance in the development of educational strategies for sports education. The relatively lower number of scholars for “Physical Education” and “Sport Pedagogy” suggests that these areas may be less explored or face greater competition within the broader academic and research communities. Moreover, the discrepancy between national and international representation may reflect the limited engagement of Italian scholars in global networks, emphasizing the necessity to increase international visibility with different collaborations.

The correlation analyses provided relevant information on the interrelationships between the various bibliometric metrics considered for the period 2020–2023. Spearman's correlation showed a significant positive relationship between total citations and total h-index (*rs* = 0.83), indicating that authors with a higher number of citations also tended to have a higher overall h-index. This strong correlation suggested that the quality and impact of scientific production, measured through the number of citations, consistently reflected in the scholars' overall h-index. This implied that academic recognition of scholars was closely tied to the impact of their publications. The high correlation between total citations and h-index supports the idea that a strong scholarly reputation and academic recognition in the field of sport pedagogy is based on impactful and well-cited research. The number of citations is a reliable measure of the quality of work produced, as it reflects the frequency with which scholars' research is cited and used by others in the field. In addition, the positive correlation indicates that the more frequently an article is cited, the more likely the author is to achieve an influential h-index, confirming the link between research visibility, quality, and academic prestige over time.

The correlation between relative citations and relative h-index (*rs* = 0.61) was also significant, indicating that even when considering only scientific output related to the three keywords, there was consistency between the number of citations received and the specific h-index for these publications. This suggested that publications focused on “Physical Education,” “Sport Pedagogy,” and “Sport Education” had a measurable and significant impact contributing to the scholars' relative h-index. In other words, sports science scholars receiving more citations on topics related to sports pedagogy also tended to have a higher relative h-index. This indicated a trend where greater attention and citation in sports pedagogy works translated into greater overall recognition, measured through the h-index. The moderate correlation between relative citations and relative h-index reinforces the idea that research specifically related to sports pedagogy has solid academic value, directly impacting scholars' professional recognition. As the papers of these scholars receive more citations in sports pedagogy, their relative h-index also increases. This highlights how focused research on specialized topics can build scholars' reputations, even if their overall citation metrics are not as high as those in broader fields.

Finally, the moderate positive correlation between relative citations and total h-index (*rs* = 0.32) indicated a relationship between the specific impact of publications in the ESS subfield of sports pedagogy and the scholars' total h-index. This meant that over time, these scholars' studies became impactful within the scientific community (Altavilla et al., 2013; D'Elia, 2020). This implied that their studies on the identified keywords had more impact compared to other general topics. However, the relationship was not as strong as those observed by other metrics. This might suggest that Italian scholars in sports pedagogy had a broader and more diversified scientific output contributing to their total h-index, in addition to publications related to the three specific keywords.

Among the potential limitations of the study, it was essential to consider specific issues associated with the Scopus search engine, including metadata errors, the presence of duplicates, or the lack of updates on certain publications, which could affect the accuracy and reliability of the results obtained through the research. Despite these limitations, the originality of the study remains a significant strength, as it addresses a gap in the literature on sports pedagogy within the Italian context and provides new insights into the visibility and impact of Italian scholars in this area. Future studies could build upon these findings by incorporating additional data sources or more refined bibliometric tools to enhance the precision of the analysis.

## 5 Conclusions

The results suggested that the scientific production of Italian scholars in the educational didactic field of ESS was significant both nationally and internationally, although with differences in the distribution of the three keywords “Physical Education,” “Sport Pedagogy,” and “Sport Education” and in international visibility. The interesting correlation between relative citations and total h-index indicated that these scholars had more impact with studies related to the identified keywords compared to other general topics. The analysis also highlighted the lack of international impact of Italian research in the educational didactic part of Motor and Sports Sciences, particularly for “Physical Education,” which had no Italian representative at the international level during the period considered. In summary, although Italian research in the education-al didactic field showed good consistency and significant impact, it was necessary to improve the visibility of Italian scholars at the international level regarding research in terms of “Physical Education.” To enhance the international impact of Italian research in the educational didactic field of ESS, it is essential to promote greater collaboration with international scholars. Additionally, strengthening interdisciplinary approaches and aligning research priorities with global trends can improve visibility and citation impact. Policymakers and institutions should support targeted funding initiatives and academic strategies to bridge the gap between national and international research influence, particularly in “Physical Education,” where representation remains limited.

## Data Availability

The original contributions presented in the study are included in the article/supplementary material, further inquiries can be directed to the corresponding author.
